# Gain-of-function mutations of *AtNHX1* suppress *sos1* salt sensitivity and improve salt tolerance in Arabidopsis

**DOI:** 10.1007/s44154-021-00014-1

**Published:** 2021-11-22

**Authors:** Isaiah Catalino M. Pabuayon, Jiafu Jiang, Hongjia Qian, Jung-Sung Chung, Huazhong Shi

**Affiliations:** 1 Department of Chemistry and Biochemistry, Texas Tech University, Lubbock, TX 79424 USA; 2 Current address: State Key Laboratory of Crop Genetics and Germplasm Enhancement, Key Laboratory of Landscaping, Ministry of Agriculture and Rural Affairs, Key Laboratory of Biology of Ornamental Plants in East China, National Forestry and Grassland Administration, College of Horticulture, Nanjing Agricultural University, Nanjing, 210095 China; 3 Current address: Department of Agronomy, Gyeongsang National University, Jinju, 52828 South Korea

**Keywords:** *sos1* suppressor, *AtHKT1*, *AtNHX1*, Gain-of-function, Salt tolerance

## Abstract

Soil salinity severely hampers agricultural productivity. Under salt stress, excess Na^+^ accumulation causes cellular damage and plant growth retardation, and membrane Na^+^ transporters play central roles in Na^+^ uptake and exclusion to mitigate these adverse effects. In this study, we performed *sos1* suppressor mutant (named *sup*) screening to uncover potential genetic interactors of *SOS1* and additional salt tolerance mechanisms. Map-based cloning and sequencing identified a group of mutants harboring dominant gain-of-function mutations in the vacuolar Na^+^/H^+^ antiporter gene *AtNHX1*. The gain-of-function variants of AtNHX1 showed enhanced transporter activities in yeast cells and increased salt tolerance in Arabidopsis wild type plants. Ion content measurements indicated that at the cellular level, these gain-of-function mutations resulted in increased cellular Na^+^ accumulation likely due to enhanced vacuolar Na^+^ sequestration. However, the gain-of-function suppressor mutants showed reduced shoot Na^+^ but increased root Na^+^ accumulation under salt stress, indicating a role of AtNHX1 in limiting Na^+^ translocation from root to shoot. We also identified another group of *sos1* suppressors with loss-of-function mutations in the Na^+^ transporter gene *AtHKT1*. Loss-of-function mutations in *AtHKT1* and gain-of-function mutations in *AtNHX1* additively suppressed *sos1* salt sensitivity, which indicates that the three transporters, SOS1, AtNHX1 and AtHKT1 function independently but coordinately in controlling Na^+^ homeostasis and salt tolerance in Arabidopsis. Our findings provide valuable information about the target amino acids in NHX1 for gene editing to improve salt tolerance in crops.

**Supplementary Information:**

The online version contains supplementary material available at 10.1007/s44154-021-00014-1.

## Introduction

Crop productivity and plant health are severely compromised by high soil salinity. Salt stress leads to disequilibrium of sodium (Na^+^) and nutritional ions in plant cell, which could result in cell death and plant destruction. Specifically, excess Na^+^ can cause disruptions in normal enzymatic activity and impair cellular metabolism. Therefore, maintenance of ion homeostasis in the cell is required for better plant health under salt stress. Previous studies have identified genes that help Na^+^ homeostasis and promote cellular growth and survival (Blumwald 2000; Hasegawa et al. 2000; Zhu 2003; Assaha et al. 2017; Basu et al. 2021). Central to these are plasma membrane and vacuolar Na^+^ transporters that directly control cellular Na^+^ concentration and subcellular Na^+^ allocation.

*SOS1* (*SALT-OVERLY-SENSITIVE1*) encodes a plasma membrane Na^+^/H^+^ antiporter, and its functional mutant *sos1* is hypersensitive to NaCl (Shi et al. 2000). Conversely, overexpression of *SOS1* increases salt tolerance by maintaining Na^+^ homeostasis under high salinity (Shi et al. 2003). SOS1 is phylogenetically related to other plasma membrane Na^+^/H^+^ antiporters such as *Schizosaccharomyces pombe* SOD2 and *Saccharomyces cerevisiae* NHA1 and mediates cellular Na^+^ efflux (Jia et al. 1992; Prior et al. 1996; Shi et al. 2000). At the whole plant level, SOS1 plays a critical role in controlling long-distance Na^+^ transport from root to shoot (Shi et al. 2002; El Mahi et al. 2019). Its activity is induced by salt stress signals that are transduced by the calcium (Ca^2+^)- dependent SOS signaling pathway. This pathway comprises of the Ca^2+^-binding protein SOS3 physically interacting with and activating the protein kinase SOS2, which, upon forming a protein kinase complex, phosphorylates and activates SOS1 by releasing its autoinhibition (Qiu et al. 2002; Zhu 2003; Quintero et al. 2011). Additionally, several other possible regulatory motifs are present in SOS1, which includes a binding motif for cyclic nucleotides and an interacting domain for the signaling protein RCD1 (Katiyar-Agarwal et al. 2006; Gong et al. 2020).

AtHKT1 is a plasma membrane transporter identified to mediate Na^+^ uptake (Rubio et al. 1995; Uozumi et al. 2000). Its importance in Na^+^ homeostasis was established by its genetic interaction with the SOS pathway genes. Loss-of-function *hkt1* alleles suppress the salt hypersensitivity of *sos1*, *sos2,* and *sos3* mutants (Rus et al. 2001). Genetic and physiological evidence supports that *AtHKT1* controls Na^+^ translocation in plants, and it also plays a role in the regulation of plant K^+^ nutrition (Rus et al. 2004). It was initially proposed that *AtHKT1* mitigates Na^+^ accumulation in shoots by recirculating Na^+^ to the roots via the phloem transport system (Maser et al. 2002; Berthomieu et al. 2003). However, *AtHKT1* was later found to selectively unload Na^+^ from xylem vessels to xylem parenchyma cells, and thus reducing Na^+^ translocation to shoots and protecting photosynthetic leaves from salinity stress (Sunarpi et al. 2005). *AtHKT1;1* was further demonstrated to independently control Na^+^ accumulation in root and Na^+^ retrieval from the xylem (Davenport et al. 2007). Specifically, overexpression of *AtHKT1;1* in the mature root stele increased salt tolerance of the transgenic Arabidopsis by reducing Na^+^ accumulation in the shoot due to increased influx of Na^+^ into stellar root cells (Møller et al. 2009). The *HKT1* system presents an evolutionally important salt sequestration mechanism in plants, as increasing number of homologs were found and characterized in other species, especially in agriculturally important monocots such as rice and wheat (Hamamoto et al. 2015; Ali et al. 2019; Gong et al. 2020). In fact, allelic variations in *HKT1* and its homologs display differences in salinity tolerance within a species (Campbell et al. 2017; Busoms et al. 2018).

The vacuolar Na^+^/H^+^ antiporter gene *AtNHX1* has been found to improve salt tolerance in Arabidopsis and tomato through overexpression (Apse et al. 1999; Zhang and Blumwald 2001). AtNHX1 is similar sequence-wise with the mammalian NHE and the yeast NHX1 Na^+^/H^+^ exchangers (Counillon and Pouyssegur 2000). AtNHX1 complements the yeast *nhx1* mutant and suppresses its Na^+^/Li^+^ sensitive phenotype, indicating that AtNHX1 functions as an endosomal Na^+^/H^+^ antiporter (Gaxiola et al. 1999; Quintero et al. 2000). More importantly, *NHX1* overexpression improved salinity tolerance in multiple plant species (Shi and Zhu 2002; Chen et al. 2007; Zhang and Shi 2013; Sahoo et al. 2016; Nguyen et al. 2019). As a vacuolar membrane Na^+^/H^+^ antiporter, AtNHX1 compartmentalizes Na^+^ to the vacuole and prevents toxic Na^+^ accumulation in the cytoplasm and aids in acclimation to salt stress (Blumwald 2000; Hasegawa et al. 2000; Gong et al. 2020). Vacuolar Na^+^ compartmentalization and the accumulation of organic solutes in the cytosol and other organelles play crucial roles in maintaining cellular water status and intracellular osmotic status (Blumwald et al. 2000). Na^+^ transport across the plasma membrane and tonoplast is dependent on the proton (H^+^) electrochemical potential established by H^+^-translocating pumps. Upregulation of H^+^-pyrophosphatase pump (H^+^-PPase) has been found to increase salt and drought tolerance in plants (Gaxiola et al. 2001; Park et al. 2005; Brini et al. 2007; Shen et al. 2015). In addition, the H^+^-pyrophosphatase AVP1 also plays a role in auxin transport and consequently auxin-dependent development (Li et al. 2005). Like *AVP1, AtNHX1* has demonstrated other physiological functions such as control of cellular pH and development. For example, a null mutation in the homolog of *AtNHX1* in morning glory (*Ipomoea nil*) disrupts the vacuolar pH of corolla cells, leading to altered flower color (Fukada-Tanaka et al. 2000; Yamaguchi et al. 2001). Other *AtNHX1* homologs in Arabidopsis and other plant species are also reported to function in regulating pH, K^+^ homeostasis, vesicle trafficking, and cell expansion, all of which have substantial importance in plant development (Rodriguez-Rosales et al. 2009; Bassil et al. 2011; Reguera et al. 2015; Qiu 2016).

In this study, we present the identification and characterization of *sos1* suppressors. Gain-of-function (dominant) mutations in *AtNHX1* and loss-of-function (recessive) mutations in *AtHKT1* that suppress the salt sensitivity of *sos1–1* were identified. These suppressor mutations prevented cytosolic Na^+^ accumulation and mitigated Na^+^ translocation to the shoot and thus conferring enhanced salt tolerance in *sos1–1*. We further demonstrated that single gain-of-function amino acid substitutions in AtNHX1 provide improved salt tolerance in Arabidopsis, which paves a new avenue for crop improvement in salt tolerance by gene editing in the future. Our findings further highlight the complex interaction among the three crucial transporters for salt tolerance in Na^+^ homeostasis and salt tolerance in plants.

## Results

### Identification of *sos1* suppressors

**Fig. 1:**
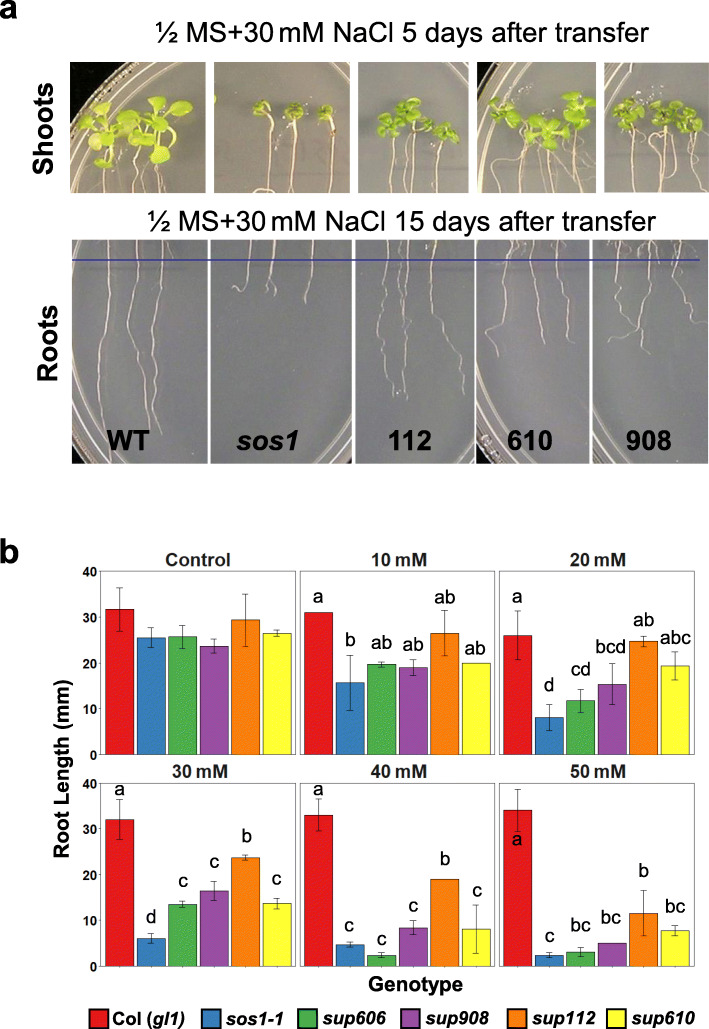
Phenotypes of *sos1* suppressor mutants. **a** Shoot and root growth of wild type (WT), *sos1–1*, and selected *sos1* suppressor mutants. Five-day-old seedlings grown in ½ MS agar media (1.2% agar) were transferred to the same media supplemented with 30 mM NaCl. **b** Quantification of the root length of WT, *sos1–1* and *sos1* suppressor mutants grown in ½ MS agar media supplemented with different concentrations of NaCl. Bars represent means, and error bars represent standard errors (*n* = 5). Statistical significance among genotypes within treatments was computed with ANOVA and Tukey’s post-hoc HSD test (*P* < 0.05) and different statistical groups are represented by letters

We performed a genetic screening for mutants suppressing the salt sensitivity of *sos1* to identify genes coordinately working with the plasma membrane Na^+^/H^+^ antiporter SOS1 in conferring salinity tolerance. The *sos1–1* allele carrying a 14 bp deletion (Shi et al. 2000) was used as the genetic background to avoid *sos1* revertant mutants and recover additional mutations suppressing *sos1–1*. From a screening of ~ 250,000 M_2_ individuals of EMS-mutagenized *sos1–1* and re-screening of putative *sos1* suppressor lines, more than 40 lines were verified to be bona fide *sos1* suppressors (designated as *sup*) and not *sos1* revertant lines since no additional mutations were found in the *sos1–1* mutant gene. Figure 1a shows the *sos1* suppression phenotype of three selected *sup* mutants. The *sup* lines showed improved growth of both shoot and root when the growing media was supplemented with 30 mM NaCl. Root length measurements indicated that the four tested *sup* lines showed differences on the capacity of suppression of *sos1–1* salt sensitivity (Fig. 1b). The *sup112* suppressor mutant displayed strongest suppression of *sos1–1* salt sensitivity in root growth, and the other three suppressors only showed clear suppression at 30 mM NaCl. However, *sup112* exhibited weaker suppression in leaves than other two suppressors and *sup610* showed better leaf growth among the tested suppressors (Fig. 1a). These results implied that multiple genes may be involved in suppressing the salt sensitivity of *sos1–1*, and these genes may have different tissue-specific modes of activity in response to salt stress.

### Gain-of-function mutations in *AtNHX1* suppress *sos1* salt sensitivity

**Fig. 2:**
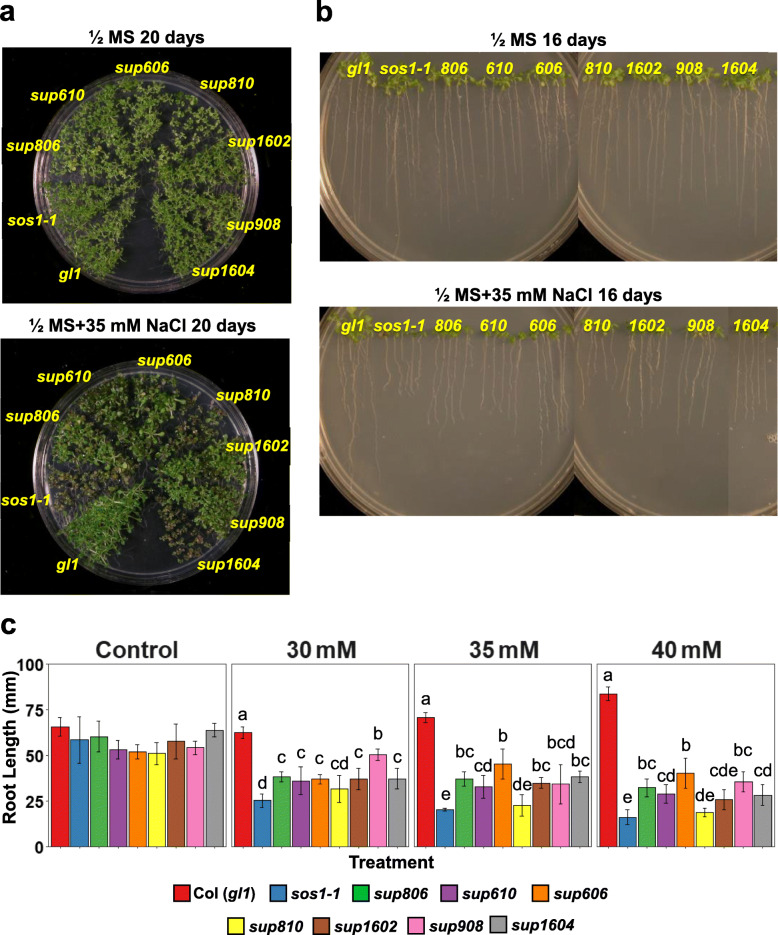
Phenotyping of the *sup* lines harboring mutations in *AtNHX1*. **a** The suppressor lines exhibit suppression of *sos1* salt sensitivity in shoot growth under salinity stress (35 mM NaCl). **b** Root growth phenotype of the *sup* lines. **c** Quantification of the root length of WT, *sos1–1* and the *sup* suppressor mutants. Bars represent means, and error bars represent standard errors (*n* = 5). Statistical significance among genotypes within treatments was computed with ANOVA and Tukey’s post-hoc HSD test (*P* < 0.05) and different statistical groups are represented by letters

**Fig. 3:**
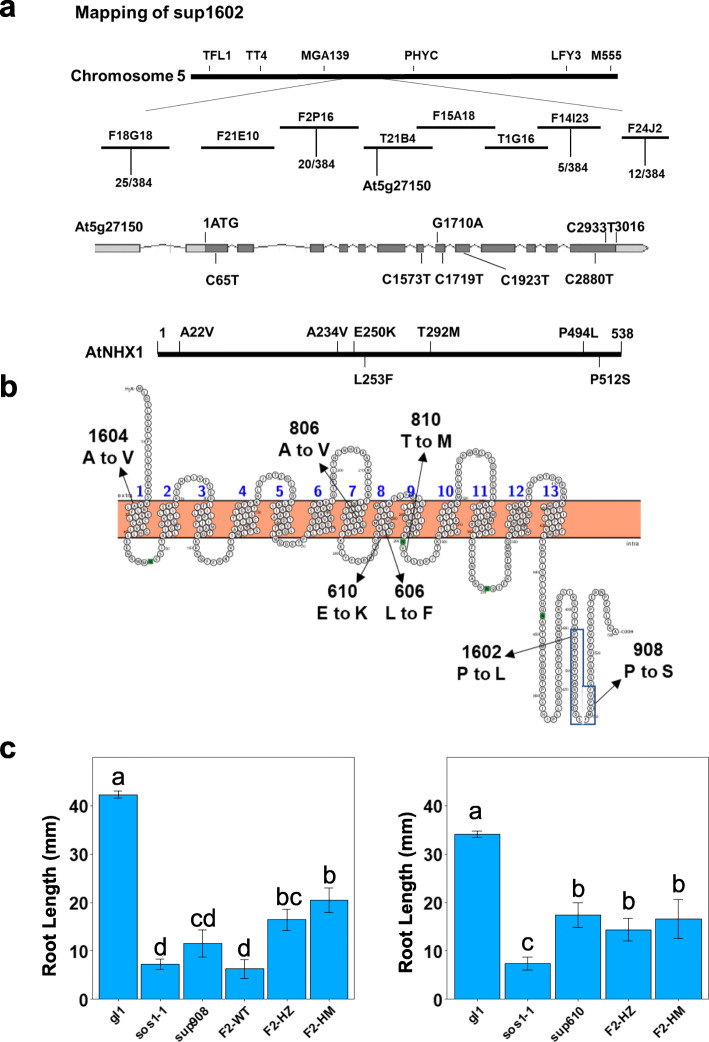
Identification of the dominant mutations in *AtNHX1* gene in the *sup* mutants. **a** Positional cloning of the *sup1602* mutant gene. The *sup* mutant alleles were identified by sequencing the *AtNHX1* gene in the *sup* mutants. Nucleotide changes and amino acid substitutions in the gene and protein of the *sup* mutants are shown. **b** Predicted secondary structure of AtNHX1 and the amino acid substitutions in the *sup* mutants. The structure was illustrated using Protter (Omasits et al. 2013). **c** The root lengths for the different genotypes indicating improved salinity tolerance in all genotypes carrying a *sup AtNHX1* allele. HZ, heterozygote, HM, homozygote. Bars represent means, and error bars represent standard errors (*n* = 5). Statistical significance was computed with ANOVA and Tukey’s post-hoc HSD test (*P* < 0.05) and different statistical groups are represented by letters

Gene mapping and sequencing revealed a group of *sup* mutants (*sup606*, *610*, *806*, *810*, *908*, *1602*, and *1604*) harboring mutations in the vacuolar Na^+^/H^+^ antiporter gene *AtNHX1*. These *sup* lines showed better growth in both shoots (Fig. 2a) and roots (Fig. 2b, c) compared to the *sos1–1* mutant under salinity stress. The suppressor mutant *sup1602* was initially chosen for positional cloning and the mutant gene was delimited to the region between the BAC clones F2P16 and F14I23 in chromosome 5 (Fig. 3a). After examining the genes within this region, the previously characterized salinity tolerance determinant gene *AtNHX1* was found in this region and was an apparent candidate for the suppressor gene. Sequencing of the *AtNHX1* gene in *sup1602* mutant revealed a heterozygous C2880T nucleotide change in the coding region (Fig. 3a, Fig. S1), which suggested that the mutation could be a dominant, gain-of-function mutation. Subsequent sequencing of the *AtNHX1* gene in all other suppressor lines identified the above-mentioned seven lines harboring mutations in *AtNHX1* gene, among which four lines had homozygous mutations and three possess heterozygous mutations (Fig. 3a, b, Fig. S1). Crosses between the homozygous mutants *sup908* and *sup610* with wild-type Col (*gl1*) revealed that both heterozygotes and homozygotes of the suppressor lines displayed suppression of the *sos1–1* salt sensitivity (Fig. 3c, Fig. S2), which further supported that the mutations in *AtNHX1* gene are dominant mutations conferring salt tolerance. The suppressor mutations in the *AtNHX1* gene resulted in amino acid substitutions in vital positions of the protein (Fig. 3b). The lines *sup1604* and *806* possess an Ala to Val change in predicted transmembrane domains, while *sup610* and *sup606* have transmembrane residue changes of Glu to Lys and Leu to Phe, respectively. The *sup810* mutant harbors an amino acid change of Tyr to Met in a loop, which alters a prospective N-glycosylation site (NVT to NVM). For *sup1602* and *908*, the mutations are situated in the C-terminal region of AtNHX1, which result in amino acid substitutions in the previously characterized calmodulin-interacting motif (Yamaguchi et al. 2005).

**Fig. 4:**
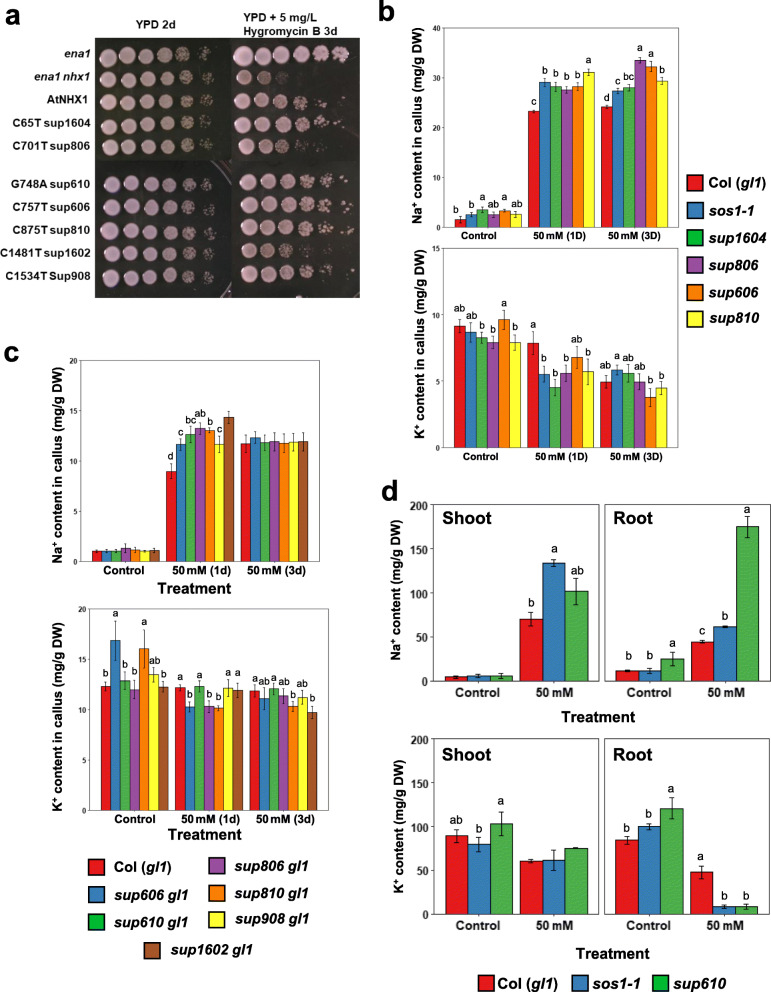
The suppressor mutations in *AtNHX1* are gain-of-function mutations. **a** Yeast complementation assay showing enhanced activity of AtNHX1 carrying the *sup* mutations. AtNHX1 activity was assessed in yeast (*Saccharomyces cerevisiae*) by its capacity conferring tolerance to hygromycin B (5 mg L^− 1^). **b**, **c** Na^+^ and K^+^ contents in the callus cells of *sup* mutants in the *sos1–1* background (**b**) and Col (*gl1*) background (**c**). Calli were sampled (*n* = 3) at one (1d) and 3 days (3d) after transferring to the agar medium containing 50 mM NaCl. Confidence intervals (CI) were used to determine statistically significant differences within treatments and letters denote different groups (*P* < 0.05). Bars represent means, and error bars represent the CI (*n* = 3). **d** Na^+^ and K^+^ contents in the shoots and roots of Col (*gl1*), *sos1–1*, and a representative *sup* mutant line, *sup610*. Bars represent means, and error bars represent standard errors (*n* ≥ 3). Statistical differences among genotypes within a treatment group was computed with ANOVA and Tukey’s post-hoc HSD test (*P* < 0.05, *n* ≥ 3) and different statistical groups are represented by letters

Gain-of-function mutations of *AtNHX1* in the suppressor mutants were further verified by heterologous expression of the mutated *AtNHX1* in yeast cells (Fig. 4a). As shown in previous studies (Gaxiola et al. 1999; Hernández et al. 2009), expression of the wild type *AtNHX1* complemented and rescued the hygromycin sensitivity of the yeast mutant with a defective tonoplast Na^+^/H^+^ antiporter ScNHX1. Compared with the wild type *AtNHX1*, expression of the *AtNHX1* suppressor variants *sup1604, 610, 606,* and *810* in yeast exhibited superior resistance to hygromycin (Fig. 4a), indicating increased activity of the variant transporters. Notably, *sup806, 1602,* and *908* did not show increased activity in the yeast cells, suggesting that the mechanisms of these alterations enhancing AtNHX1 function in plant may not exist in yeast. This is especially true for *sup1602* and *908* which harbor mutations in the regulatory C-terminal region of the protein known to interact with and be inhibited by binding with AtCaM15 (Yamaguchi et al. 2005). This regulatory mechanism might be unique to plants or at least absent in yeast. Our findings also suggest that the suppressor mutations improve the efficacy of the AtNHX1 transporter through different mechanisms. In addition, genetic analysis using the T-DNA knockout mutant of *AtNHX1* further supported the gain-of-function feature of the suppressor mutations. The double mutant *sos1nhx1* displayed more salt sensitivity than the single mutant *sos1* (Fig. S3), which is opposite to the enhanced salt tolerance of *sos1* by the gain-of-function suppressor mutations (Fig. 2).

In accordance with the gain-of-function mutations in *AtNHX1* in the *sup* mutants, the callus cells of the *sup* lines accumulated more Na^+^ than the *sos1–1* mutant after 50 mM NaCl treatment for 3 days (Fig. 4b), which is likely due to enhanced AtNHX1 activity and thus increased Na^+^ sequestration into the vacuoles of the *sup* lines. The K^+^ contents in *sos1–1* and *sup* lines were lower than the wild type after salt treatment for 1 day, perhaps mainly due to the effects of *sos1* mutation on K^+^ uptake. Enhanced Na^+^ accumulation by the gain-of-function mutations in AtNHX1 was further confirmed by the analysis of Na^+^ and K^+^ in the callus cells of the AtNHX1 gain-of-function mutants in the wild type (Col (*gl1*)) background (Fig. 4c). The results showed that all the gain-of-function mutants accumulated significantly higher Na^+^ in the callus cells than the wild type after salt treatment for 1 day. However, the Na^+^ contents in all lines are comparable after 3 days salt treatment, suggesting a limit in the capacity for vacuolar Na^+^ sequestration (Fig. 4c).

Measurements of ion contents at the whole plant level provide insight to the possible mechanism of how the *AtNHX1* variants suppress *sos1* salt sensitivity (Fig. 4d). Under salinity stress, the *sos1–1* mutant shoot accumulated higher Na^+^ than wild type, while the suppressor mutant *sup610* had reduced Na^+^ accumulation compared with *sos1–1* mutant. In the roots, *sup610* showed the highest Na^+^ content among the three lines, while *sos1–1* had significantly higher Na^+^ than the wild type under salt stress condition. These results suggest that the enhanced AtNHX1 activity in *sup610* helps prevent the translocation of Na^+^ into the photosynthetic shoot tissues by accumulation of Na^+^ in the roots, likely through the sequestration of Na^+^ into the vacuoles of the root cells, thus reducing the rate of Na^+^ movement from root to shoot. The K^+^ content in shoot is comparable among the three lines, while both *sos1–1* and *sup610* displayed drastically decreased K^+^ accumulation in roots when compared with the wild type.

### Mutations in the AtNHX1 C-terminal region disrupts the inhibitory binding site for AtCaM15

**Fig. 5:**
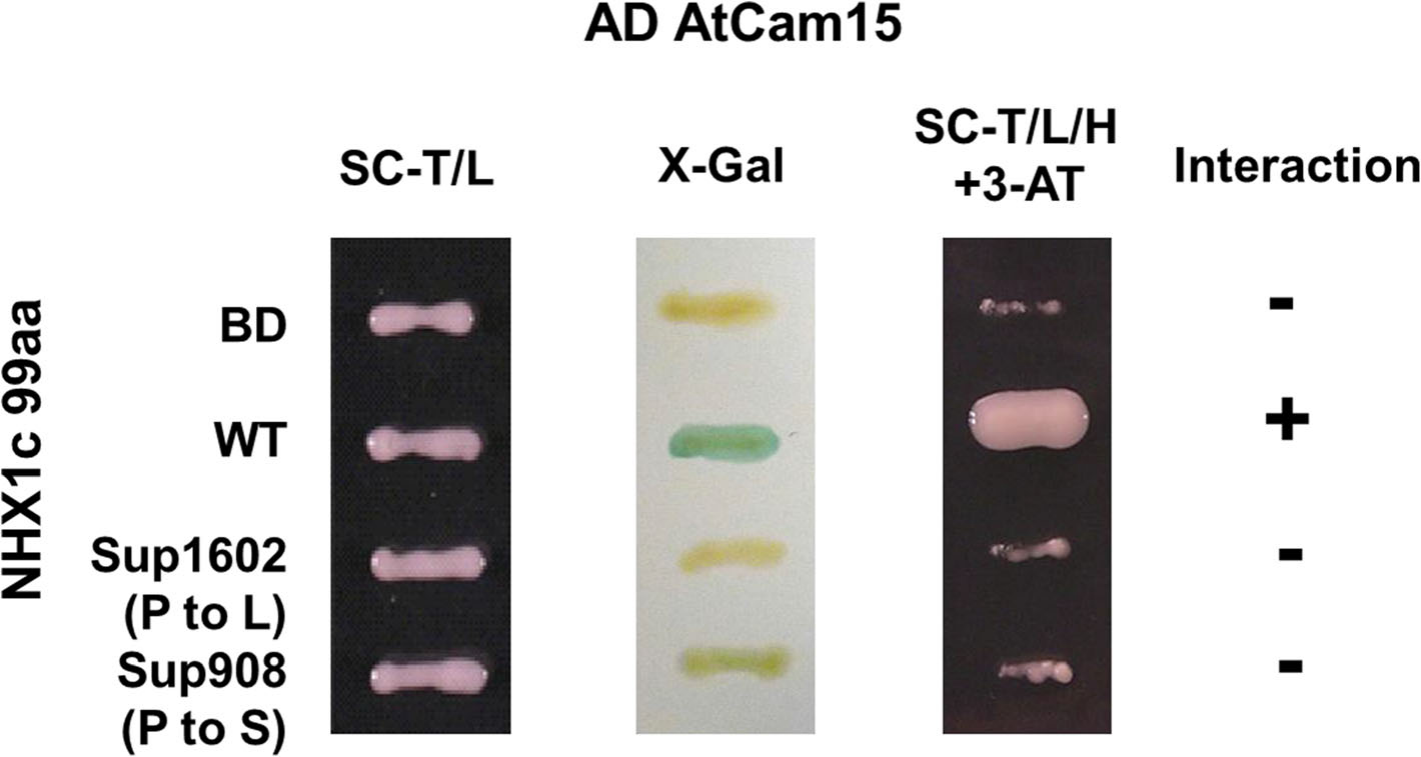
Protein interaction assay between AtCaM15 and the C-terminal region of the wild type and *sup* alleles of AtNHX1. Yeast two-hybrid assay was conducted to determine the interactions. The C-terminal (99 aa) region of AtNHX1 was used as the bait fused with the Gal4 binding domain, while AtCaM15 was fused with the Gal4 activation domain. Positive interaction (+) was indicated by the blue color resulting from the metabolism of X-gal and vigorous growth under reductive synthetic media (SC-T/L/H + 3-AT)

AtCaM15, a calmodulin-like protein, has been shown to inhibit the activity of AtNHX1 by binding to its C-terminal region in a Ca^2+^- and pH-dependent manner (Yamaguchi et al. 2005). Among the seven identified suppressor lines, two *sup* mutants, *sup1602* and *sup908*, harbor amino acid substitutions in the regulatory C-terminal domain (Fig. 3b). A yeast two-hybrid interaction assay was conducted to determine the binding activity of AtCaM15 to these AtNHX1 variants (Fig. 5). As expected, the wild type AtNHX1 C-terminal region (99 amino acids) interacted with AtCaM15 as indicated in the X-Gal transactivation test and synthetic deficient media growth test. In contrast, the gain-of-function AtNHX1 variants *sup1602* and *908* could not interact with AtCaM15. This result clearly indicates the disruption of the CaM-interacting domain in these two AtNHX1 variants. Since binding of AtCaM15 suppresses the transport activity of AtNHX1, disruption of the interaction in these two *sup* lines releases the inhibitory activity of AtCaM1 and enhances Na^+^ transport activity across the vacuolar membrane. We further tested whether a loss-of-function mutation in *AtCaM15* could also suppress *sos1* salt sensitivity since the gene knockout of *AtCaM15* is supposed to eliminate its binding to and inhibition of AtNHX1. However, the *sos1cam15* double mutant did not show enhanced salt tolerance but rather exhibited even slightly increased salt sensitivity (at 40 mM NaCl) compared to the *sos1* single mutant (Fig. S4), These results suggest that, while AtCaM15 is important in the regulation of AtNHX1 activity, there may be other calmodulins or calmodulin-like proteins that have overlapping function with AtCaM15 in regulating AtNHX1. Additionally, it also implies that *AtCaM15* plays a role in salt tolerance independent of *AtNHX1*.

### Mutations in *AtHKT1* led to suppression of *sos1* salt sensitivity

**Fig. 6:**
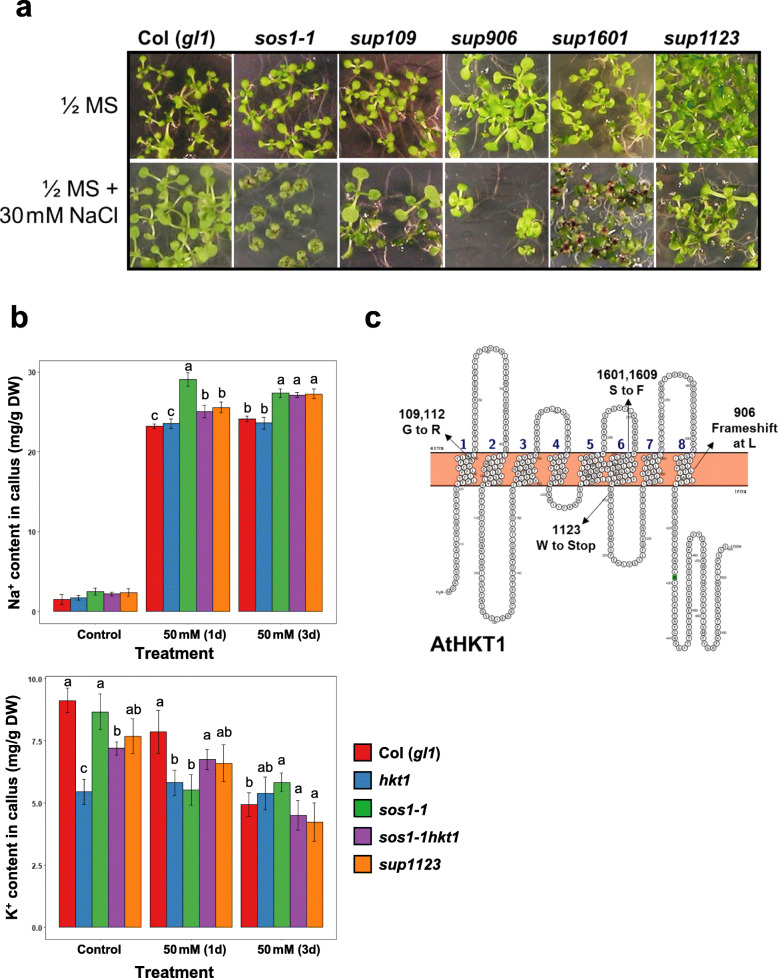
Identification of *sup* lines with loss-of-function mutations in *AtHKT1*. **a** *sos1* suppression phenotype of the *sup* mutants harboring mutations in *AtHKT1* gene. **b** Na^+^ and K^+^ contents in the callus cells of Col-gl1, *hkt1*, *sos1–1*, *sos1hkt1* and *sup1123*. Calli were sampled (*n* = 3) at one (1d) and 3 days (3d) after transferring to the agar medium containing 50 mM NaCl. Confidence intervals were computed and used to determine different statistical groups within treatments, which are denoted by the letters above each bar representing the means (*P* < 0.05, *n* = 3). **c** Secondary structure of AtHKT1 and the amino acid substitutions in the *sup* mutants. The secondary structure was illustrated using Protter (Omasits et al. 2013)

Previous studies have shown that mutations in the *AtHKT1* gene suppress the salt sensitivity of *sos* mutants (Rus et al. 2004). We therefore sequenced the *AtHKT1* gene, including the promoter region, in all other *sup* mutants and identified six lines (*sup109, 112, 906, 1123, 1601,* and *1609*) harboring mutations in *AtHKT1* gene (Fig. 6a**;** *sup1609* not shown). Na^+^ and K^+^ content in callus cells showed that, after one-day salt treatment, the *sos1–1* mutant accumulated more Na^+^ than the wild type and *athkt1* T-DNA mutant clearly suppressed Na^+^ accumulation in the *sos1–1* mutant. While the *hkt1* single mutant did not show effects on Na^+^ accumulation, mutation in *hkt1* alone clearly affected K^+^ uptake under both control and salinity stress conditions (Fig. 6b). These results indicate that *AtHKT1* controls Na^+^ and perhaps also K^+^ uptake at the cellular level. The *sos1* suppressor *sup1123* identified in this study behaved similarly with the previously characterized *sos1–1hkt1* mutant harboring a T-DNA insertion in the *AtHKT1* gene (Rus et al. 2004; Baek et al. 2010; Fig. 6b), further supporting that these *sup* lines harbor knockout mutations in the *AtHKT1* gene. The six lines represented four unique mutations in AtHKT1 (Fig. 6c). The *sup109* and *112* mutants had a substitution of Gly to Ala at the 36th amino acid residue, *sup1601* and *1609* had a substitution of Ser to Phe at the 278th residue, *sup1123* had a premature stop codon at the 309th codon, and *sup906* had a single nucleotide deletion causing frame shaft after the 403rd codon. The single amino acid substitutions (*sup109,112, 1601,* and *1609*) resulting in the loss of function could provide valuable information for understanding of the structure-function relations in AtHKT1 transporter.

### AtHKT1 loss-of-function and AtNHX1 gain-of-function additively suppress *sos1* salt sensitivity

**Fig. 7:**
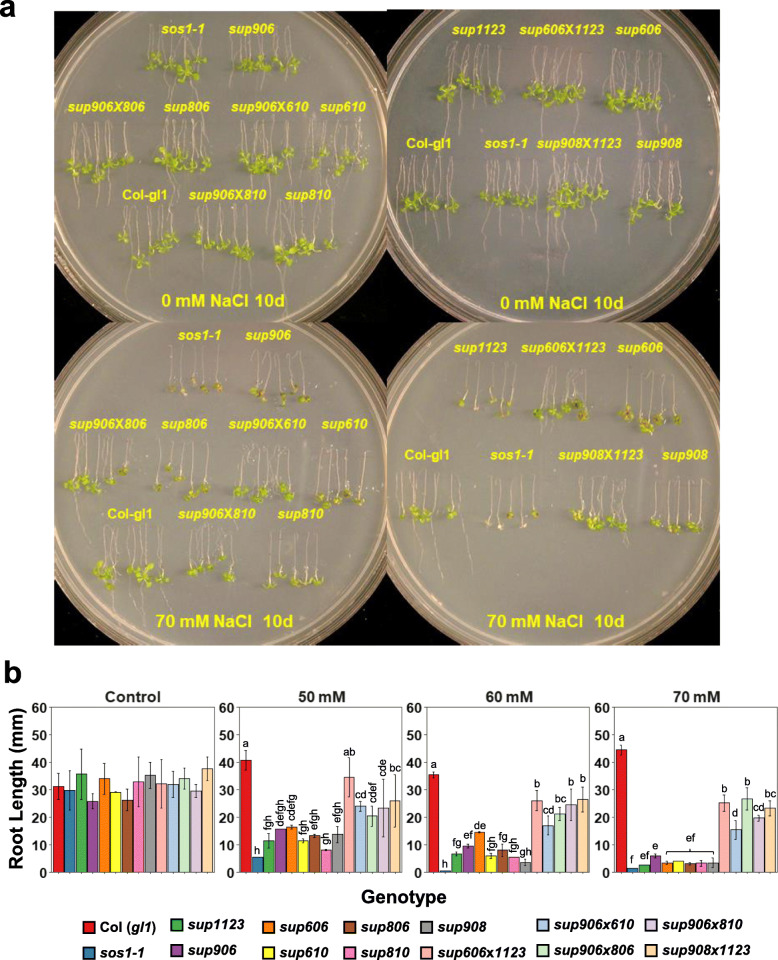
Additive suppression effects of *sup athkt1* and *sup AtNHX1* mutants. **a** Root bending assay of wild type, *sos1–1*, *sup athkt1*, *sup AtNHX1*, and double suppressor mutants. The double suppressor mutants were generated by genetic crosses between the *athkt1* and *AtNHX1 sup* lines. Five-day-old seedlings grown vertically in ½ MS agar (1.2%) medium were transferred to the same medium supplemented with different concentrations of NaCl, and the plates were put upside down for root bending assay. **b** Quantitative measurement of the root lengths of different genotypes in response to different concentrations of NaCl. Bars represent means, and error bars represent standard errors (*n* ≥ 3). Statistical significance among genotypes within a treatment group was computed with ANOVA and Tukey’s post-hoc HSD test (*P* < 0.05) and different statistical groups are represented by letters

Since AtHKT1 controls the entry of Na^+^ into the cell and AtNHX1 controls the vacuolar influx of Na^+^, combining the loss-of-function mutation in *AtHKT1* and gain-of-function mutation in *AtNHX1* is expected to be additive in suppression of *sos1* salt sensitivity. To test this hypothesis, combinations of different *sup* lines for *AtHKT1* and *AtNHX1* were genetically crossed, and double suppressor mutants were generated. Salt sensitivity assay showed that, at 70 mM NaCl, single *sup* mutants phenotypically resembled the *sos1–1* mutant. In contrast, the double *sup* lines clearly showed better salt tolerance than the single *sup* lines in both root and shoot growth (Fig. 7a). Root length measurements showed a steady decrease in single *sup* lines along with increasing salt concentrations (Fig. 7b). At 70 mM NaCl, single *sup* lines did not show *sos1* suppression phenotype in root growth. In contrast, double *sup* lines displayed stronger root growth compared to the tested single *sup* lines from 50 mM NaCl to 70 mM NaCl in addition to healthier shoots. These findings suggest a possibility of further improving plant salt tolerance by combining different salt tolerance mechanisms such as reducing cellular Na^+^ influx and increasing vacuolar Na^+^ sequestration to mitigate cytoplasmic Na^+^ toxicity.

### Gain-of-function mutations in *AtNHX1* improve salt tolerance in the wild type background

**Fig. 8:**
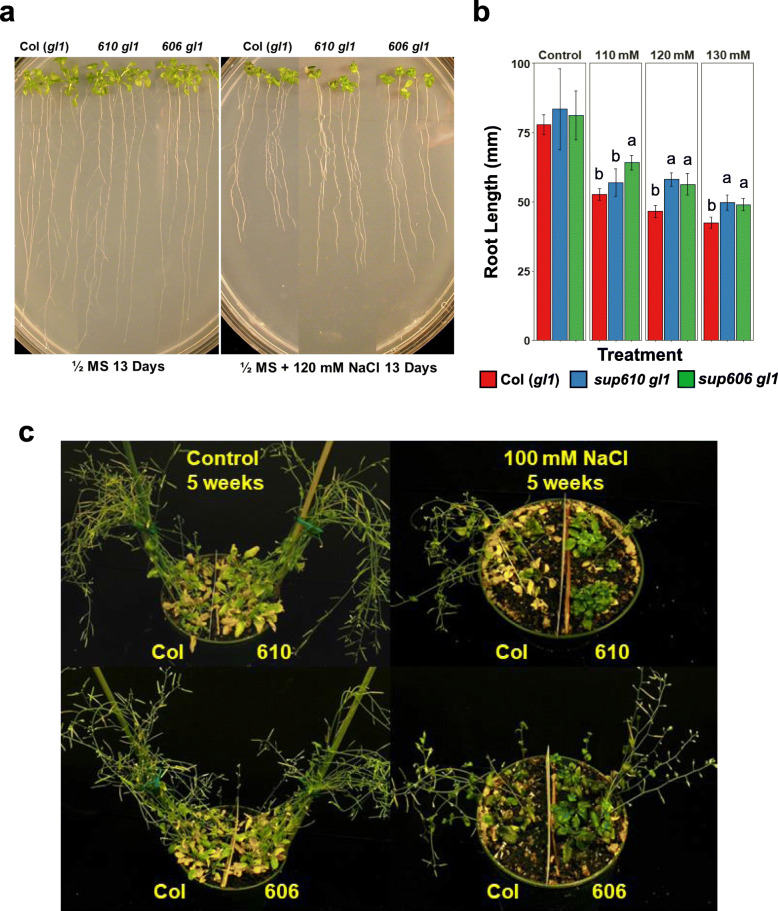
Gain-of-function *AtNHX1* variants conferring increased salt tolerance in the wild-type background. **a** Root growth assay of the wild type Col (*gl1*) and the single *AtNHX1* gain-of-function alleles 606 and 610 in Col (*gl1*) background. The single gain-of-function *AtNHX1* mutant alleles were obtained by crossing the *sup* mutants with Col (*gl1*) background and determined by genotyping from the F_2_ population. **b** Quantitative measurement of the root lengths of Col (*gl1*) and the single gain-of-function mutants. Bars represent means, and error bars represent standard errors (*n* ≥ 3). Statistical significance among genotypes within a treatment group was computed with ANOVA and Tukey’s post-hoc HSD test (*P* < 0.05) and different statistical groups are represented by letters. **c** Soil-grown plants showing enhanced salt tolerance of the *AtNHX1* gain-of-function mutant lines compared to the wild type

To determine the feasibility of using the identified *AtNHX1* gain-of-function variants for improvement of salt tolerance in normal plant genotypes, *AtNHX1 sup* mutants were crossed with Col (*gl1*) and single *AtNHX1* gain-of-function mutants without the *sos1–1* mutation were identified from the resulting F_2_ populations. Under high salinity treatment (110 mM to 130 mM NaCl), plants harboring the *sup610* or *606 AtNHX1* allele displayed slightly better root growth (Fig. 8a, b). These plants consistently showed significantly higher mean root lengths than Col (*gl1*) plants especially at 120 mM and 130 mM NaCl treatments. When plants were grown in soil to maturity under salt treatment (1/8 MS + 100 mM NaCl), the increased salt tolerance conferred by the *AtNHX1* variant alleles was evident (Fig. 8c). Col (*gl1*) plants displayed more shoot wilting and bleached leaves, while lines with the *sup606* and *sup610* alleles exhibited far greener leaves under this treatment. However, the plants with the *sup610* allele displayed delayed bolting compared to those with the *sup606* allele under salt stress, which shows normal bolting and floral development like the wild type.

## Discussion

Salinity stress is a widespread problem hampering crop productivity. Readjustment of cellular ion homeostasis is one of the crucial processes for plants to cope with salt stress (Zhu 2003; Basu et al. 2021). Salt tolerance is partly based on the capacity of the plant to compartmentalize toxic Na^+^ ions and thus reduce Na^+^ accumulation in the cytoplasm, where most of the cell’s metabolic processes occur (Munns and Tester 2008; Munns and Gilliham 2015). Therefore, enhancing the mechanisms that increase the capacity of plant cells to reduce cytoplasmic Na^+^ concentrations improves plant salinity tolerance. At a whole plant level, tissue-specific accumulation of Na^+^ is also an important mechanism to mitigate salt stress. For example, allelic variants of *OsHKT1;1* in rice offer different capacities in excluding Na^+^ in roots (Campbell et al. 2017). In this study, we identified the genetic and physiological interactions between three membrane transporters, SOS1, HKT1, and NHX1 that are crucial for salt stress response and tolerance in Arabidopsis. We found that both loss-of-function of *AtHKT1* and gain-of-function of *AtNHX1* additively suppress *sos1* salt sensitivity. Our findings indicate that these three transport systems act independently but coordinately to modulate cellular Na^+^ compartmentalization and plant salt stress response.

*SOS1* is a vital plasma membrane Na^+^ transporter for salt exclusion by transporting Na^+^ out of the cell and thus preventing excess cytoplasmic Na^+^ accumulation in Arabidopsis (Shi et al. 2000; Shi et al. 2002). Its importance in maintaining ion homeostasis has been reported in other plant species (Olías et al. 2009; Razzaque et al. 2013; Feki et al. 2014; El Mahi et al. 2019; Wang et al. 2021). In this study, we found that two routes of lowering cellular Na^+^ accumulation counteract the loss of SOS1 function, i.e., prevention of cellular Na^+^ influx through AtHKT1 and enhancement of vacuolar Na^+^ compartmentalization by AtNHX1.

*HKT1* was initially discovered to be a K^+^ influx transporter whose K^+^ transport activity was blocked and replaced with Na^+^ transport at high salinity conditions (Rubio et al. 1995). It has been hypothesized that at the whole plant level, *HKT1* is important for lowering Na^+^ concentration in the xylem sap to prevent Na^+^ accumulation in photosynthetic tissues (Sunarpi et al. 2005). Thus, loss of this control for shoot Na^+^ accumulation leads to salt hypersensitivity of the *hkt1* mutant. However, it has been shown that disruptive mutations of *hkt1* can suppress *sos1, sos2,* and *sos3* salt sensitivity (Rus et al. 2004). This implies that *hkt1* is independent of the *SOS* pathway. This notion is also supported in our study, wherein the *sup* mutants carrying loss-of-function alleles of *AtHKT1* improve the tolerance of *sos1* mutant (Fig. 6). This may be simply due to the reduction of Na^+^ influx, leading to better survival under mild salt stress. Indeed, at higher Na^+^ concentrations and prolonged exposure to salt stress, these *sup* lines do not confer any advantage of salt tolerance. In addition, *hkt1* mutations only confer salt tolerance in the *sos1* background but not wild type background, suggesting a complex interaction between these two genes. Nonetheless, Baxter et al. (2010) showed that a weak allele of *HKT1* in accessions growing in coastal regions confers increased salt tolerance. This further indicates that HKT1 is an evolutionarily important Na^+^ transporter in plant adaptation to saline environment and could be used as a marker for salt tolerance breeding in crops.

A previous study by Hernández et al. (2009) identified *NHX1* mutants improving tolerance to ion toxicity in yeast by increasing its selectivity to K^+^ over Na^+^ and Li^+^. This suggests that gain-of-function mutations in NHX1 could improve salt tolerance. Through *sos1* suppressor screening, we also identified a group of dominant and gain-of-function mutations in *AtNHX1* conferring increased salt tolerance in both *sos1* and wild type background (Figs. 2, 8, Fig. S3). These gain-of-function mutations could be classified into two types: 1) transmembrane domain mutants, and 2) regulatory domain mutants. The mutations in the transmembrane domains (TMs) of the three mutants (i.e., *sup806, 610, 606*) are concentrated around the predicted TM-7 and TM-8 regions and may directly affect the binding and transport of Na^+^ and H^+^. The *sup1604* mutation is located in the predicted TM-1, which is further from TM-7 and TM-8 in the secondary structure. However, TM-1 might be a part of the catalytic region in the 3D structural context. Interestingly, both *sup1604* and *sup806* have an Ala to Val substitution, which is a subtle change in terms of amino acid properties but nonetheless increases its transport activity. This indicates that the length of the side chain of these two amino acids may play a role in Na^+^ and H^+^ transport by AtNHX1. Regardless, the enhanced activity of these variants was evidenced in the transformed yeast and in callus cells, especially for *sup610* and *606* (Fig. 4a, c). The enhanced transport activity improves vacuolar Na^+^ sequestration, leading to better plant salt tolerance.

The mutation in *sup810* is on a possible N-glycosylation site in the loop between TM-9 and TM-10 and thus may alter a regulatory mechanism. Protein glycosylation has been shown to be important for protein folding (Nagashima et al. 2018) and implicated in plant development and stress response (Trinidad et al. 2020). While the glycosylation of AtNHX1 at this site is unknown, the *sup810* mutation increases AtNHX1 activity as evidenced by the yeast complementation assay (Fig. 4a). This suggests that glycosylation, if present at this site in AtNHX1, is a common inhibitory mechanism in plant and yeast. Glycosylation might block the ion binding and transport site of AtNHX1 since the NVT site is at the TMs possibly forming its ion transport channel (Fig. 3b). On the other hand, the mutations in *sup908* and *1602* are on the C-terminal tail that has been shown to bind with and inhibited by AtCaM15 in a pH-dependent manner (Yamaguchi et al. 2005). Binding of AtCaM15 leads to a decrease in the Na^+^ transporter activity of AtNHX1, while maintaining its K^+^ transporter activity. It was hypothesized that this interaction represses AtNHX1 activity under normal growth conditions, and dissociation activates AtNHX1 under salt stress. Although this inhibitory mechanism may have some importance under ideal growing conditions, such an inhibition is clearly inadequate when vacuolar Na^+^ sequestration is required to reduce cytoplasmic Na^+^ under salinity stress conditions. Thus, the mutant alleles of *sup908* and *1602* which are unable to be inhibited by AtCaM15 have elevated activity to deal with salt stress. While the full extent of the trade-offs associated with this type of mutations is not yet known, the identified gain-of-function mutations may be more beneficial than overexpression in improving salt tolerance in plants because the AtNHX1 variants resemble the wild type AtNHX1 and do not carry the same metabolic burden as overexpression. This hypothesis is supported by our observation that the *sup* alleles driven by the constitutive overexpressing promoter (CaMV 35S), although providing enhanced salt tolerance in the *sos1–1* mutant, did not suppress the salt sensitivity of *sos1* to the same degree as the identified *sup* lines (Fig. S5). In a system such as a plant under salt stress, limited metabolic productivity is a major constraint for survival, especially from the processes that consume ATP (Wu et al. 2016; Pabuayon et al. 2021).

The results presented here indicate the independent action of AtSOS1, AtNHX1, and AtHKT1 in maintaining ion homeostasis under salinity stress. It has been proposed that these three transporters act in support of each other to minimize the accumulation of Na^+^ in photosynthetic or metabolically active tissues (Zhang and Shi 2013; Gong et al. 2020). Cellular Na^+^ exclusion provided by AtSOS1 may be the primary mechanism of maintaining Na^+^ homeostasis, which is supported by the extremely salt sensitive phenotype of the *sos1* mutants (Shi et al. 2000). In addition, the activities of AtHKT1 and AtNHX1, which both transport Na^+^ to prevent cytosolic Na^+^ accumulation, play crucial roles in cellular Na^+^ homeostasis. At the whole plant level, the accumulation of Na^+^ in parenchyma cells via the activity of AtHKT1 lowers Na^+^ concentration in the root xylem sap, leading to reduced Na^+^ translocation to shoots. AtNHX1 further reduces salt stress damage by compartmentalizing Na^+^ to the vacuoles and reducing Na^+^ accumulation in shoots. Therefore, these three transporters independently while synergistically function in maintaining Na^+^ homeostasis at both cellular and whole plant levels. Similar strategies in maintaining ion homeostasis have been reported for other plant species, including halophytes (Horie et al. 2012; Zhang et al. 2017). This indicates the conservation of these mechanisms in salt stress response in different plant species, while variant transporters may partly account for the capability of salinity tolerance.

The identified gain-of-function mutations in AtNHX1 in this study are mostly conserved in other important crop species. The alignment of the NHX1 homologs in cotton (*Gossypium hirsutum*), rice (*Oryza sativa*), maize (*Zea mays*), and soybean (*Glycine max*) with AtNHX1 showed conservation in five of the seven identified amino acids (Fig. S6). For the mutation in *sup806* (Ala to Val at the 227th residue), all the other species examined have a natural Val residue. The only mutated residue that had a strong difference in AtNHX1 compared to its homologs is the mutation in *sup1604* (Ala to Val at the 22nd residue), as other species have a consensus Ser residue instead of an Ala residue in AtNHX1. This result implies the importance of the mutation sites in NHX1 function for Na^+^ transport. In addition, the conservation of these alleles points to the possible target sites of NHX1 transporter for gene editing in major crops. Recent advances in gene editing technologies, particularly CRISPR-Cas, have become increasing prevalent in creating superior crop genotypes (Arora and Narula 2017; Steinwand and Ronald 2020). The improvements may be geared towards abiotic stresses, such as drought, salt, or heat, or towards pathogen and disease resistance (Zaidi et al. 2018; Zafar et al. 2019). However, the approach requires a target gene and alleles from which the edits can be patterned to. The results from our study provide viable alleles of *NHX1* that can be created by gene editing in major crops, as majority of the functional mutations identified are well-conserved in major crop species.

To conclude, we have identified loss-of-function alleles of *AtHKT1* and gain-of-function alleles of *AtNHX1* confer salt tolerance in *sos1* mutant. Notably, the identified gain-of-function mutations in AtNHX1 are all single amino acid substitutions resulting in enhanced functionalities. These mutations provide direct target sites for gene editing of NHX1 transporters in crops for improved salt tolerance. Moreover, these mutations provide valuable information for better understanding of the structure-function relation of Na^+^/H^+^ antiporter and its activity regulation. Future studies could use protein structure modeling to uncover the mechanistical importance of the identified amino acids of AtNHX1 in Na^+^ and H^+^ transport, which will assist computational design of superior NHX1 transporter for genetic modifications and improvement of salt tolerance in crops.

## Materials and methods

### Plant materials, *sos1* suppressor screening, and genetic mapping

The *sos1–1* mutant seeds were treated with 0.3% ethyl methanesulfonate (EMS) for 15 h for mutagenesis and the M_2_ population of the mutagenized seeds were generated for *sos1* suppressor (named as *sup*) screening (Kim et al. 2006). The M_2_ seedlings were grown in ½ Murashige and Skoog (MS) agar medium (Phytotechnology Laboratories, KS) containing 30 mM NaCl and the seedlings showing better growth than *sos1–1* seedlings were selected as putative *sos1* suppressor mutants. After rescreening, the suppressor mutants were further confirmed by sequence verification of the *sos1–1* mutation in the suppressor mutants. For map-based cloning, the *sos1* suppressor mutant *sup1602* was crossed with the *sos1* T-DNA knockout alleles (ET11830/GT7225) in the Landsberg ecotype background. The F_1_ plants were selfed and the *sos1* suppressor mutants were selected from the segregating F_2_ population by the root-bending assay (Wu et al. 1996). The F_2_ recombinant lines were used to map the *sos1* suppressor gene using Col/Landsberg erecta polymorphic markers. The *sup1602* mutation was mapped between the markers CER451900 and CER 449133, and the *AtNHX1* gene in this region was considered as the prime candidate gene*.* The *AtNHX1* gene was sequenced in *sup1602* and all other *sup* mutants to determine the suppressor mutations. The inheritance pattern of the suppressor phenotype was determined through the crossing of *sup908* and *610* mutants with the *sos1–1* genotype. F_2_ lines from the crosses were genotyped using cleaved amplified polymorphic sequence (CAPS) markers. The *sup908* allele was identified through cleavage by Sau3AI, which does not cleave the wild type *AtNHX1* allele. The *sup610* allele was identified through non-cleavage by MnlI, which cleaves the wild type *AtNHX1* allele.

### Genetic interaction analysis of *sos1*, *atnhx1*, and *atcam15* mutants

To determine the genetic interaction between the loss-of-function of *AtNHX1* and *SOS1*, the *nhx1* T-DNA line was crossed with the *sos1–1* mutant and *nhx1sos1–1* double mutant was identified by genotyping. The presence of the T-DNA insertion was determined by PCR using the primers 5′-gtttctcctaagtaccttgcttgg-3′ and 5′-caaaggtatgcctgtttcatcga-3′, together with the T-DNA left border primer (5′-cattttataataacgctgcggacatctac-3′) (Apse et al. 2003). The T-DNA line for *AtCaM15* (SALK_143508C) was identified from the SALK sequence-indexed T-DNA insertion collections (Alonso et al. 2003). The flanking T-DNA insertion primers 5′-atgagctgcgacggaggca-3′ and 5′-tcaaccccaagcattatcaaacg-3′ were used together with the primer LBb1 (5′-gcgtggaccgcttgctgcaact-3′) for mutant identification and homozygosity analysis. The *cam15* T-DNA mutant was crossed with *sos1–1* mutant and *cam15sos1–1* doble mutant was generated for genetic analysis.

### Yeast complementation assay

The *AtNHX1* open reading frame was amplified via PCR using the primers 5′-ccggaattcgcatgttggattctctagtgtcgaaactg-3′ and 5′-ccgctcgagtcaagccttactaagatcaggaggg-3′. The amplified fragment was inserted into EcoRI and XhoI sites of pENTR™1A (Invitrogen, Carlsbad, CA). Site-directed mutagenesis was conducted to generate the mutations in *AtNHX1* corresponding to the identified mutations in the suppressor lines by using the primers shown in Table S1, following manufacturer’s instructions (Stratagene, La Jolla, CA). The *AtNHX1* ORF was recombined into the yeast vector pAG426GPD-ccdB (Addgene) using the Gateway LR clonase II Enzyme Mix (Invitrogen, Carlsbad, CA). The constructs were transformed into the yeast strain *ena1nhx1* via the lithium acetate/single-stranded carrier DNA/polyethylene glycol method (Gietz and Woods 2002). The yeast complementation assay was done as described by Yamaguchi et al. (2005).

### Salt stress tolerance assay

Seeds of the mutants and wild-type Col (*gl1*) were sterilized for 15 min with 20% Clorox bleach plus 0.1% Triton X-100 solution and were washed at least five times with sterilized water. The seeds were stratified at 4 °C for 2 days and sown on ½ MS medium with 1.5% sucrose and 1.2% agar. The seeds were then germinated vertically at 23 °C under a long-day cycle (16 h daylight/8 h night). Five-day-old seedlings were transferred onto ½ MS medium containing the designated NaCl concentration, 1.5% sucrose and 1.2% agar. Root lengths were measured from at least three plants (*n* ≥ 3). For soil salinity stress testing, ten-day-old seedlings grown in ½ MS agar medium were transferred to 10 cm pots with soil. Seven days after transplanting, the plants were watered indirectly (from the bottom) with 1/8 MS medium containing 100 mM NaCl weekly.

### Yeast two-hybrid interaction assay

Interaction assays were conducted as described in Yamaguchi et al. (2005) with modifications. Briefly, the DNA fragment corresponding to the 99-aa region at the C-terminal end of AtNHX1 (and *sup1602/908 AtNHX1* alleles) was amplified and cloned to the bait vector pDEST-GBKT7. Meanwhile, wild type *AtCaM15* cDNA was cloned to the prey vector pDEST-GADT7. The bait and prey constructs were transformed into *Saccharomyces cerevisiae* strain Y190 and grown in synthetic media without tryptophan and leucine (SC-T/L; control), and synthetic media without tryptophan, leucine, histidine and supplemented with 25 mM 3-AT (3-amino-1,2,4-triazole, Sigma; SC-T/L/H; selective). X-gal staining (*LacZ* reporter assay) was used to further confirm the positive interaction. Positive interaction was indicated by blue color in the X-gal staining assay and growth in the selective medium (Jiang et al. 2013).

### Measurement of Na^+^ and K^+^ content

Callus formation was induced from the mutants and wild-type seeds following the method described in Wu et al. (1996). The calli were transferred to callus induction medium containing 50 mM NaCl for salt stress treatment. The calli were then collected and dried in an 80 °C oven for at least 2 days and weighed. For ion content measurement in plants, 7-day-old seedling grown in agar medium were transferred to a hydroponic culture system for salt treatment and collection of shoot and root samples (Baek et al. 2010). The shoot and root samples were dried at 80 °C for at least 2 days. The dried samples (*n* ≥ 3) were digested in 3 mL concentrated HNO_3_ overnight followed by digestion at 100 °C until the solution clarified. The sample solution was diluted by 2.5% nitric acid. Na^+^ and K^+^ contents were measured with an atomic absorption spectrometer (Model 2380; Perkin-Elmer, Norwalk, CT).

### Statistical analysis, sequence alignments, and protein transmembrane structure visualization

Statistical analyses for the physiological parameters were conducted with R v4.0.4 using the ‘agricolae’ package (R Core Team 2020; de Mendiburu and de Mendiburu 2019). Tukey’s HSD post-hoc tests following analysis of variance (ANOVA) tests were performed to discern differences between different sample groups in each parameter tested. Confidence intervals were also computed in some datasets to provide evidence of statistical significance. Sequences for AtHKT1 and AtNHX1 homologs from different species were gathered from UniProt (The UniProt Consortium 2020). Protein transmembrane structure visualization was conducted through Protter (Omasits et al. 2013) and alignments were conducted using Clustal Omega (Sievers and Higgins 2014).

## Supplementary Information

**Additional file 1: Table S1.** Primers used for site-directed mutagenesis of *AtNHX1*. **Fig. S1.** Sequencing chromatogram of the identified *sup AtNHX1* mutants. The zygosity of the *sup* mutants were identified using their respective sequencing chromatograms. Arrows indicate the mutated nucleotides. **Fig. S2.** Genotype and phenotype segregation in the F_2_ of the genetic crosses between *sos1–1* and the *sup908* and *610* mutants, verifying the dominant inheritance pattern of the mutations. The inserts show the genotypes of the F_2_ seedlings identified with cleaved amplified polymorphic sequence (CAPS) markers. The *sup908* allele is identified through its cleavage by Sau3AI, which is absent in the wild type, while the *sup610* allele is identified through its non-cleavage by MnlI. Root lengths of individual seedlings are shown with color-coded bars according to their zygosity for their *AtNHX1* allele. **Fig. S3.** Growth phenotype of the *sos1nhx1* double mutant in response to salt stress. **a** Root growth assay of wild type Col (*gl1*), *sos1–1* and *sos1nhx1*. The *sos1nhx1* double mutant was generated by crossing *sos1–1* with the T-DNA insertion *atnhx1* mutant allele. **b** Quantitative measurement of root lengths of these three genotypes in response to different concentrations of NaCl. Bars represent means, and error bars represent standard errors (*n* ≥ 3). Statistical significance among genotypes within treatments was computed with ANOVA and Tukey’s post-hoc HSD test (*P* < 0.05) and different statistical groups are represented by letters. **Fig. S4.** Growth phenotype of *sos1cam15, sup1602,* and *sup908* in response to salt stress. The *sos1cam15* double mutant was obtained by crossing *sos1–1* with the T-DNA insertion mutant *cam15*. The suppressor mutants *sup1602* and *908* were used as controls showing suppression of *sos1–1* salt sensitivity, while the *cam15* mutation did not suppress *sos1–1* salt sensitive phenotype. **Fig. S5.** Overexpression of the *sup* alleles showed less suppression of *sos1–1* salt sensitivity than the native *sup* alleles. **a** Root growth assay of wild type, *sos1–1*, *sup* mutants and *sup*-OE seedlings. The *sup*-OE genotypes are the transgenic plants overexpressing the *AtNHX1* alleles in the *sos1–1* genetic background using the 35S promoter driven the ORF of the *AtNHX1* alleles. *AtNHX1* ORF cloning and site-directed mutagenesis to generate the *sup AtNHX1* alleles were described in “Materials and Methods”. The ORFs of *AtNHX1* alleles were recombined into the plant expression vector pEarleyGate 100 (Earley et al. 2006), and the constructs were transformed into *sos1–1* mutant to generate *sup*-OE plants using the floral dip method (Clough and Bent 1998). Homozygous transgenic lines were selected for salt sensitivity test. **b** Quantitative measurement of the root lengths. Bars represent mean root lengths, and error bars represent standard errors (*n* = 5). Statistical significance among genotypes within a treatment group was computed with ANOVA and Tukey’s post-hoc HSD test (*P* < 0.05, *n* = 5) and different statistical groups are represented by letters. **Fig. S6.** Partial sequence alignment of NHX1 homologs in Arabidopsis and major crop species. NHX1 homologs in major crop species (maize, rice, soybean, and cotton) were aligned and compared to the Arabidopsis AtNHX1 protein. Residues wherein mutations were identified in the *sup* lines are highlighted. Residues in yellow are not conserved in the other crop species compared to Arabidopsis, while residues in green are conserved in all species shown.

## Data Availability

The materials used in this study will be available for research upon request.
