# Supplementary material for: Gain-of-function mutations of AtNHX1 suppress sos1 salt sensitivity and improve salt tolerance in Arabidopsis
Source: Stress Biol. 2021 Nov 22;1(1):14. doi: 10.1007/s44154-021-00014-1 (PMC10441915; doi:10.1007/s44154-021-00014-1)
Supplement: Supplementary file 1 — Additional file 1: Table S1. Primers used for site-directed mutagenesis of AtNHX1. Fig. S1. Sequencing chromatogram of the identified sup AtNHX1 mutants. The zygosity of the sup mutants were identified using their respective sequencing chromatograms. Arrows indicate the mutated nucleotides. Fig. S2. Genotype and phenotype segregation in the F2 of the genetic crosses between sos1–1 and the sup908 and 610 mutants, verifying the dominant inheritance pattern of the mutations. The inserts show the genotypes of the F2 seedlings identified with cleaved amplified polymorphic sequence (CAPS) markers. The sup908 allele is identified through its cleavage by Sau3AI, which is absent in the wild type, while the sup610 allele is identified through its non-cleavage by MnlI. Root lengths of individual seedlings are shown with color-coded bars according to their zygosity for their AtNHX1 allele. Fig. S3. Growth phenotype of the sos1nhx1 double mutant in response to salt stress. a Root growth assay of wild type Col (gl1), sos1–1 and sos1nhx1. The sos1nhx1 double mutant was generated by crossing sos1–1 with the T-DNA insertion atnhx1 mutant allele. b Quantitative measurement of root lengths of these three genotypes in response to different concentrations of NaCl. Bars represent means, and error bars represent standard errors (n ≥ 3). Statistical significance among genotypes within treatments was computed with ANOVA and Tukey’s post-hoc HSD test (P < 0.05) and different statistical groups are represented by letters. Fig. S4. Growth phenotype of sos1cam15, sup1602, and sup908 in response to salt stress. The sos1cam15 double mutant was obtained by crossing sos1–1 with the T-DNA insertion mutant cam15. The suppressor mutants sup1602 and 908 were used as controls showing suppression of sos1–1 salt sensitivity, while the cam15 mutation did not suppress sos1–1 salt sensitive phenotype. Fig. S5. Overexpression of the sup alleles showed less suppression of sos1–1 salt sensitivity than the nativ [file 44154_2021_14_MOESM1_ESM.pdf]

SUPPLEMENTARY MATERIALS

Gain-of-function mutations of *AtNHX1* suppress *sos1* salt sensitivity and improve salt tolerance in Arabidopsis

Isaiah Catalino M. Pabuayon[1†], Jiafu Jiang[1,2†], Hongjia Qian[1], Jung-Sung Chung[1,3], Huazhong Shi[1*]

[1] Department of Chemistry and Biochemistry, Texas Tech University, Lubbock, TX 79424

*Author for Correspondence: huazhong.shi@ttu.edu; Tel: +1-806-834-7214

[†] These authors contributed equally to this work

[2] Current address: State Key Laboratory of Crop Genetics and Germplasm Enhancement, Key Laboratory of Landscaping, Ministry of Agriculture and Rural Affairs, Key Laboratory of Biology of Ornamental Plants in East China, National Forestry and Grassland Administration, College of Horticulture, Nanjing Agricultural University, Nanjing 210095, China

[3] Current address: Department of Agronomy, Gyeongsang National University, Jinju 52828, South Korea

Table S1. Primers used for site-directed mutagenesis.

***sup* Mutant**	**Primer Name**	**Primer Sequence (5' to 3')**
sup1604	c65t	5'-atcacgcttctgtggttgtgttgaatctctttgttgc-3'
	c65t_antisense	5'-gcaacaaagagattcaacacaaccacagaagcgtgat-3'
sup806	c701t	5'-gcaaccggtctgataagtgtgtatgttatcaagaagcta-3'
	c701t_antisense	5'-tagcttcttgataacatacacacttatcagaccggttgc-3'
sup610	g748a	5'-ggaaggcactcaactgaccgaaaggttgccctta-3'
	g748a_antisense	5'-taagggcaacctttcggtcagttgagtgccttcc-3'
sup606	c757t	5'-ctgaccgagaggttgcctttatgatgcttatggcg-3'
	c757t_antisense	5'-cgccataagcatcataaaggcaacctctcggtcag-3'
sup810	c875t	5'-ccattacacatggcacaatgtaatggagagctcaag-3'
	c875t_antisense	5'-cttgagctctccattacattgtgccatgtgtaatgg-3'
sup1602	c1481t	5'-cttcttgacacggctcactcgaaccgtgc-3'
	c1481t_antisense	5'-gcacggttcgagtgagccgtgtcaagaag-3'
sup908	c1534t	5'-ttgatgactccttcatgcgatccgtctttggagg-3'
	c1534t_antisense	5'-cctccaaagacggatcgcatgaaggagtcatcaa-3'

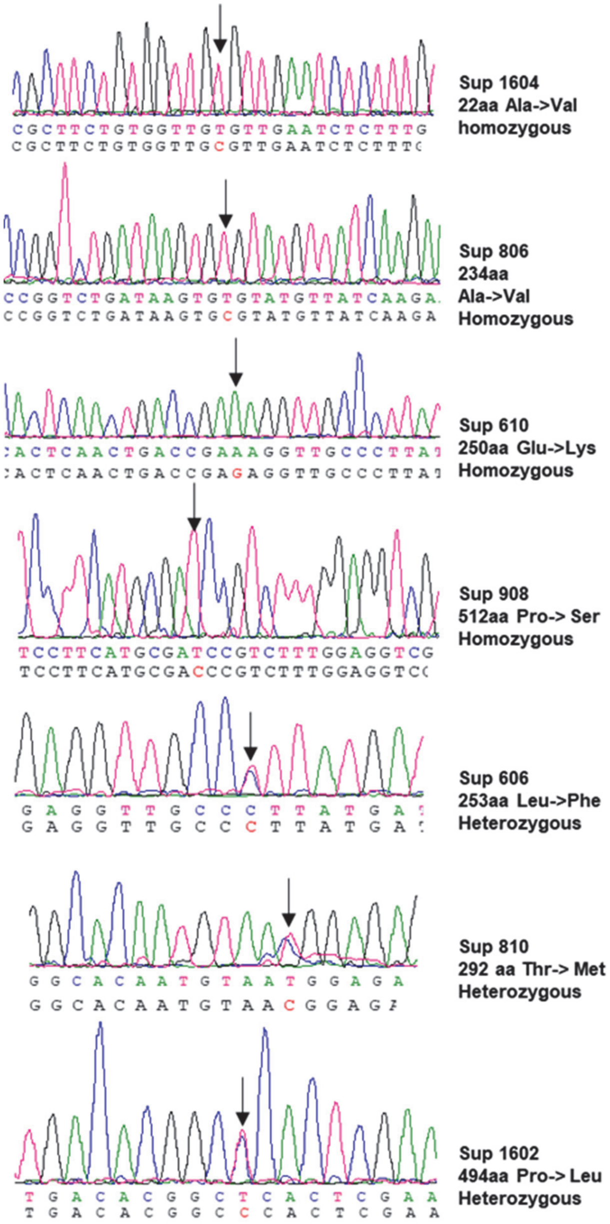

Fig. S1. Sequencing chromatogram of the identified *sup AtNHX1* mutants. The zygosity of the *sup* mutants were identified using their respective sequencing chromatograms. Arrows indicate the mutated nucleotides.

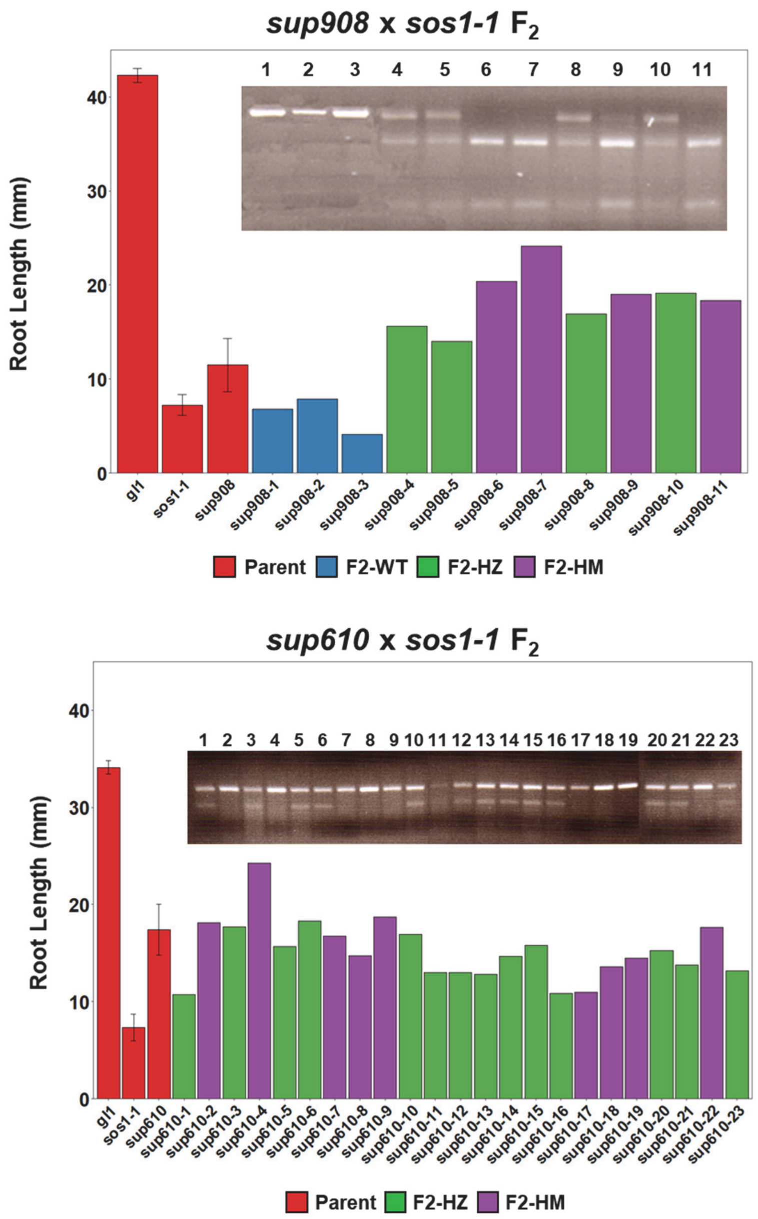

Fig. S2. Genotype and phenotype segregation in the F_2 of the genetic crosses between *sos1-1* and the *sup908* and *610* mutants, verifying the dominant inheritance pattern of the mutations. The inserts show the genotypes of the F_2 seedlings identified with cleaved amplified polymorphic sequence (CAPS) markers. The *sup908* allele is identified through its cleavage by Sau3AI, which is absent in the wild type, while the *sup610* allele is identified through its non-cleavage by MnlI. Root lengths of individual seedlings are shown with color-coded bars according to their zygosity for their *AtNHX1* allele.

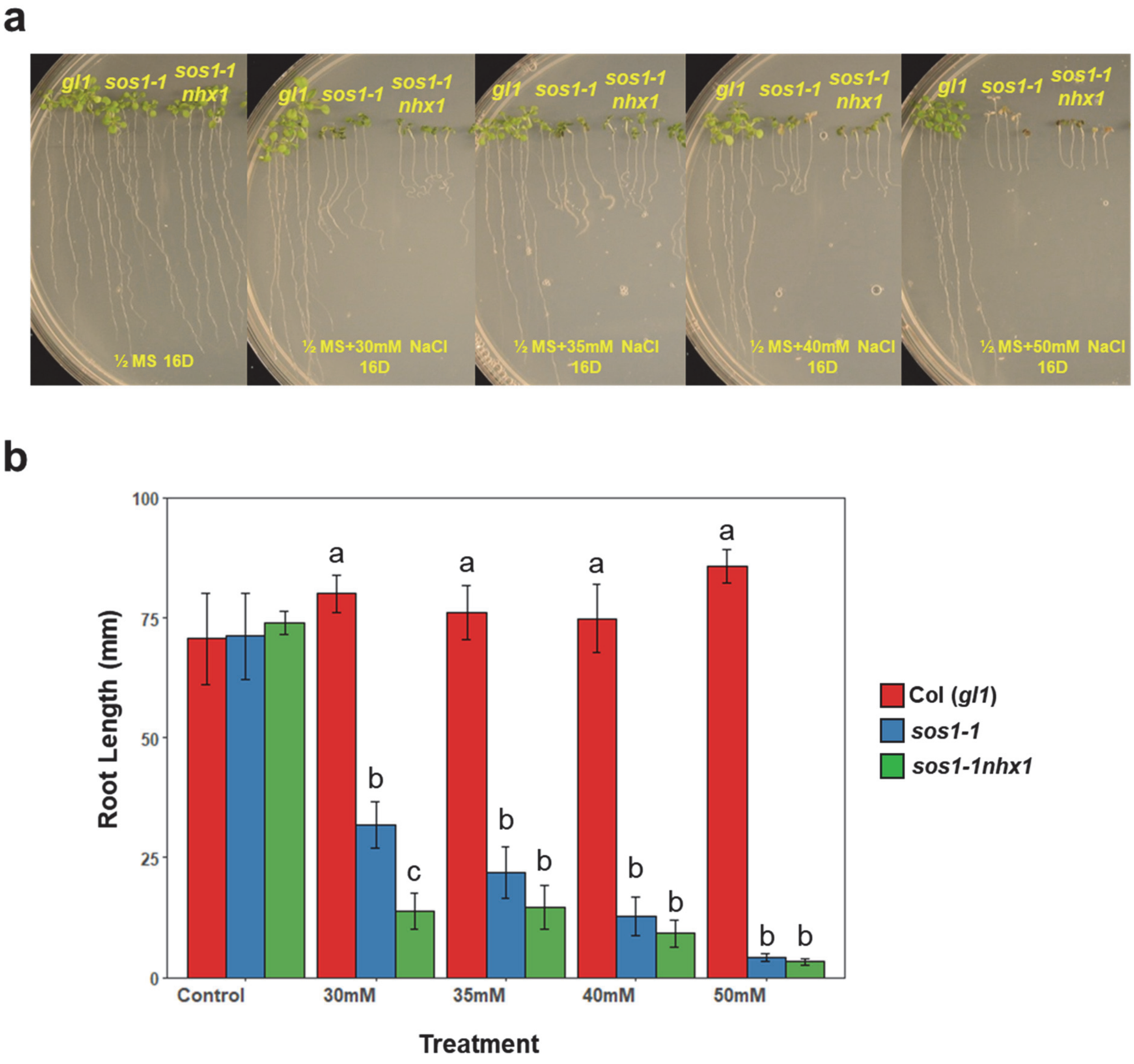

Fig. S3. Growth phenotype of the *sos1nhx1* double mutant in response to salt stress. a Root growth assay of wild type Col (*gl1*), *sos1-1* and *sos1nhx1*. The *sos1nhx1* double mutant was generated by crossing *sos1-1* with the T-DNA insertion *atnhx1* mutant allele. **b** Quantitative measurement of root lengths of these three genotypes in response to different concentrations of NaCl. Bars represent means, and error bars represent standard errors (n ≥ 3). Statistical significance among genotypes within treatments was computed with ANOVA and Tukey's post-hoc HSD test ($P < 0.05$) and different statistical groups are represented by letters.

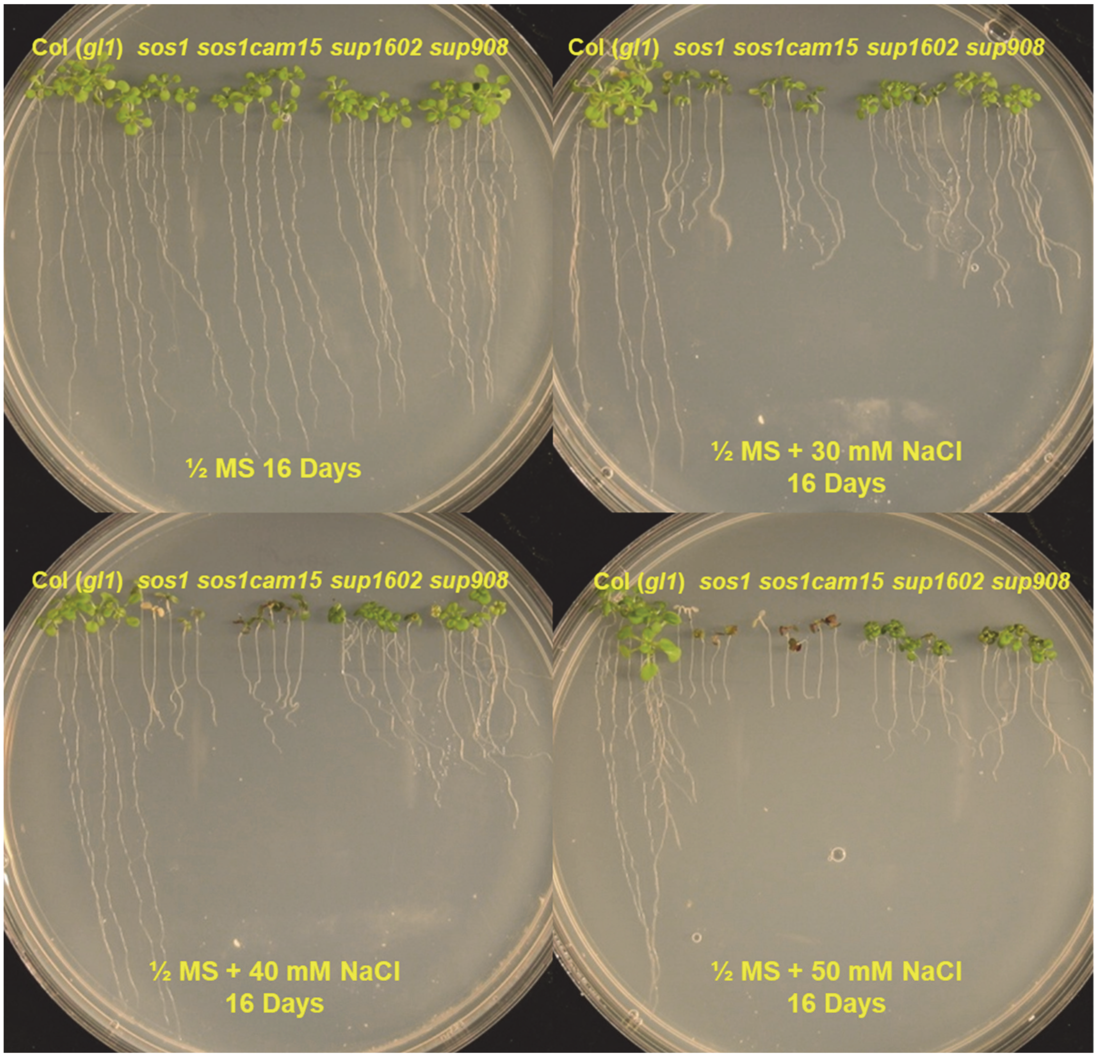

Fig. S4. Growth phenotype of *sos1cam15, sup1602,* and *sup908* in response to salt stress. The *sos1cam15* double mutant was obtained by crossing *sos1-1* with the T-DNA insertion mutant *cam15*. The suppressor mutants *sup1602* and *908* were used as controls showing suppression of *sos1-1* salt sensitivity, while the *cam15* mutation did not suppress *sos1-1* salt sensitive phenotype.

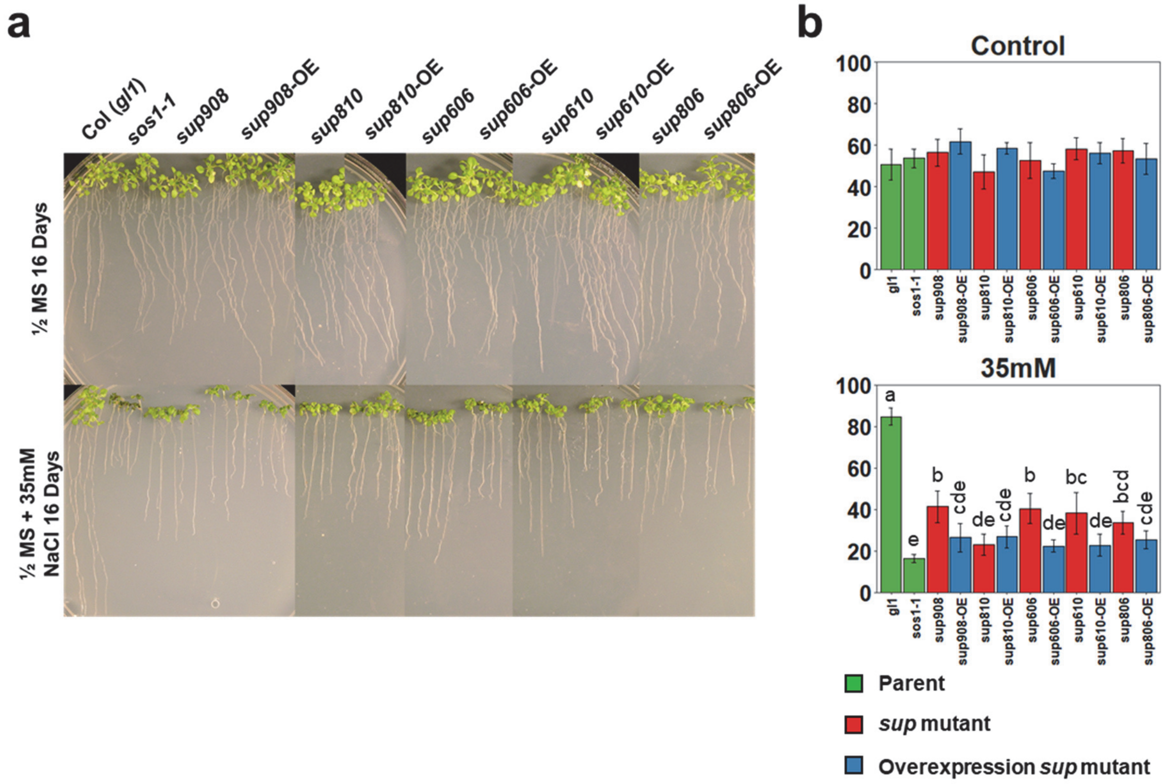

Fig. S5. Overexpression of the *sup* alleles showed less suppression of *sos1-1* salt sensitivity than the native *sup* alleles. a Root growth assay of wild type, *sos1-1*, *sup* mutants and *sup*-OE seedlings. The *sup*-OE genotypes are the transgenic plants overexpressing the *AtNHX1* alleles in the *sos1-1* genetic background using the 35S promoter driven the ORF of the *AtNHX1* alleles. *AtNHX1* ORF cloning and site-directed mutagenesis to generate the *sup AtNHX1* alleles were described in “Materials and Methods”. The ORFs of *AtNHX1* alleles were recombined into the plant expression vector pEarleyGate 100 (Earley et al. 2006), and the constructs were transformed into *sos1-1* mutant to generate *sup*-OE plants using the floral dip method (Clough and Bent 1998). Homozygous transgenic lines were selected for salt sensitivity test. **b** Quantitative measurement of the root lengths. Bars represent mean root lengths, and error bars represent standard errors (n = 5). Statistical significance among genotypes within a treatment group was computed with ANOVA and Tukey’s post-hoc HSD test ($P < 0.05$, n = 5) and different statistical groups are represented by letters.

```
                                                              sup1604
Zm00001eb330030_P001 (Maize)        MGLGVVAELVRLGVLSSTSDHASVVSINLFVALLCACIVLGHLLEENRWVNESITALIIG     60
Os07t0666900-01  (Rice)             --MGMEVAAARLGALYTTSDYASVVSINLFVALLCACIVLGHLLEENRWVNESITALIIG     58
Glyma.10g158700.1.p (Soybean)       -MVFEISSVVSKLQTLSTSDHASVVSMNLFVALLCGCIVLGHLLEENRWMNESITALLIG     59
Gohir.A11G237700.1 (Cotton)         MVAPQLAAVFTKLQTLSTSDHASVVSMNIFVALLCACIVIGHLLEENRWMNESITALIIG     60
sp|Q68KI4|NHX1_ARATH (Arabidopsis)  ----MLDSLVSKLPSLSTSDHASVVALNLFVALLCACIVLGHLLEENRWMNESITALLIG     56
                                                    :***:****::*:******.***:*********:*******:**

                                                                                   sup806
Zm00001eb330030_P001                VFGEGVVNDATSVVLFNALQNFDLNHIDVAVVLNFLGNFCYLFVSSTLLGVFTGLLSAYI     240
Os07t0666900-01                     VFGEGVVNDATSIVLFNALQNFDLVHIDAAVVLKFLGNFFYLFLSSTFLGVFAGLLSAYI     238
Glyma.10g158700.1.p                 VFGEGVVNDATSVVLFNAIQSFDLNQIDSSIAVHFLGNFLYLFIASTMLGVLTGLLSAYI     239
Gohir.A11G237700.1_UTX-TM1_v2.1     VFGEGVVNDATSVVLFNAIQSFDLVNTSPRILLEFIGSFLYLFLASTMLGVIVGLVSAYI     240
sp|Q68KI4|NHX1_ARATH                VFGEGVVNDATSVVVFNAIQSFDLTHLNHEAAFHLLGNFLYLFLLSTLLGAATGLISAYV     236
                                    ************:*:***:*.*** : .    ..::*.* ***: **:**. .**:***:

                                           sup610 sup606                                sup810
Zm00001eb330030_P001                IKKLYIGRHSTDREVALMMLMAYLSYMLAELLDLSGILTVFFCGIVMSHYTWHNVTESSR     300
Os07t0666900-01                     IKKLYIGRHSTDREVALMMLMAYLSYMLAELLDLSGILTVFFCGIVMSHYTWHNVTESSR     298
Glyma.10g158700.1.p                 IKKLYIGRHSTDREVALMMLMAYLSYMLAELCYLSGILTVFFCGIVMSHYTWHNVTESSR     299
Gohir.A11G237700.1_UTX-TM1_v2.1     IKKLYFGRHSTDREFALMMLMAYLSYIMAELFYLSGILTVFFCGIVMSHYTWHNVTESSR     300
sp|Q68KI4|NHX1_ARATH                IKKLYFGRHSTDREVALMMLMAYLSYMLAELFDLSGILTVFFCGIVMSHYTWHNVTESSR     296
                                    *****:********.***********::***  ***************************
                                                         sup1602            sup908
Zm00001eb330030_P001                QGSDIETGSAQIVRPSSLRMLLSKPTHTVHYYWRKFDDALMRPMFGGRGFVPFSPGSPTE     532
Os07t0666900-01                     QGSDLEST-TNIVRPSSLRMLLTKPTHTVHYYWRKFDDALMRPMFGGRGFVPFSPGSPTE     529
Glyma.10g158700.1.p                 QESEVDIDGHDIHRPSSIRALLTTPTHTVHRLWRKFDDAFMRPVFGGRGFVPVEPGSPTE     539
Gohir.A11G237700.1_UTX-TM1_v2.1     QDSFD-DSLIGVHRPNSIRALLTTPAHTVHYYWRKFDNAFMRPMFGGRGFVPFVPGSPTE     533
sp|Q68KI4|NHX1_ARATH                SFIEP-SGNHNVPRPDSIRGFLTRPTRTVHYYWRQFDDSFMRPVFGGRGFVPFVPGSPTE     529
                                    .          : **.*:* :*: *::***  **:**:::***:********. ******

Zm00001eb330030_P001                QSVHDGR--- 539
Os07t0666900-01                     QSHGGR---- 535
Glyma.10g158700.1.p                 RNGHQWR--- 546
Gohir.A11G237700.1_UTX-TM1_v2.1     RSEPNLPQWQ 543
sp|Q68KI4|NHX1_ARATH                RNPPDLSKA- 538
                                    :.
```

Fig. S6. Partial sequence alignment of NHX1 homologs in Arabidopsis and major crop species. NHX1 homologs in major crop species (maize, rice, soybean, and cotton) were aligned and compared to the Arabidopsis AtNHX1 protein. Residues wherein mutations were identified in the *sup* lines are highlighted. Residues in yellow are not conserved in the other crop species compared to Arabidopsis, while residues in green are conserved in all species shown.
